# The *Bradyrhizobium japonicum* exporter ExsFGH is involved in efflux of ferric xenosiderophores from the periplasm

**DOI:** 10.1371/journal.pone.0296306

**Published:** 2024-01-02

**Authors:** Alasteir Ong, Mark R. O’Brian

**Affiliations:** Department of Biochemistry, Jacobs School of Medicine and Biomedical Sciences, The University at Buffalo, Buffalo, New York, United States of America; East Carolina University Brody School of Medicine, UNITED STATES

## Abstract

The gram-negative bacterium *Bradyrhizobium japonicum* can take up structurally dissimilar ferric siderophores from the environment (xenosiderophores) to meet its nutritional iron requirements. Siderophore-bound iron transported into the periplasm is reduced to the ferrous form by FsrB, dissociated from the siderophore and the free ion is then transported into the cytoplasm by the ferrous iron transporter FeoAB. Here, we identified the RND family exporter genes *exsFG* and *exsH* in a selection for secondary site suppressor mutants that restore growth of an *fsrB* mutant on the siderophores ferrichrome or ferrioxamine. The low level of radiolabel accumulation from ^55^Fe-labeled ferrichrome or ferrioxamine observed in the *fsrB* mutant was restored to wild type levels in the *fsrB exsG* mutant. Moreover, the *exsG* mutant accumulated more radiolabel from the ^55^Fe-labeled siderophores than the wild type, but radiolabel accumulation from inorganic ^55^Fe was similar in the two strains. Thus, ExsFGH exports siderophore-bound iron, but not inorganic iron. The rescued *fsrB* exsG mutant required *feoB* for growth, indicating that ExsFGH acts on those siderophores in the periplasm. The *exsG* mutant was more sensitive to the siderophore antibiotic albomycin than the wild type, whereas the *fsrB* mutant was more resistant. This suggests ExsFGH normally exports ferrated albomycin. *B*. *japonicum* is naturally resistant to many antibiotics. The *exsG* strain was very sensitive to tetracycline, but not to six other antibiotics tested. We conclude that ExsFGH is a broad substrate exporter that is needed to maintain siderophore homeostasis in the periplasm.

## Introduction

Iron is an essential nutrient, but it has low bioavailability because it is predominantly in the ferric (Fe^3+^) state in aerobic environments. Many microorganisms secrete into the environment low-molecular-weight molecules called siderophores, which chelate Fe^3+^ with a high affinity. The ferric siderophore complex is then re-taken up by the microorganism to fulfill its nutritional iron requirement. The paradigm of ferric siderophore transport in Gram-negative bacteria consists of an outer membrane transporter, a periplasmic shuttle protein, and an inner membrane ABC transporter [1,2]. Siderophores are structurally diverse, and these transport proteins show high selectivity for one or a few structurally related siderophores.

*Bradyrhizobium japonicum* USDA110 is a gram-negative bacterium that exists free-living in soil or as an endosymbiont of the soybean plant. *B*. *japonicum* does not synthesize siderophores, but it can take up a wide range of structurally dissimilar siderophores synthesized from different microorganisms to support growth [3–5]. They are referred to as xenosiderophores in this context. *B*. *japonicum* expresses five selective outer membrane ferric siderophore receptors that can accommodate those siderophores, as well as a heme receptor [5,6].

Unlike *E*. *coli*, siderophore selectivity is limited to the outer membrane in *B*. *japonicum*, which lacks selective periplasmic and inner membrane components [5]. As a result, single base pair mutations in outer membrane receptor genes that allow transport of the synthetic iron chelator EDDHA (ethylenediamine-N,N′-bis(2-hydroxyphenylacetic acid)) are sufficient to confer growth on ferric EDDHA as an iron source [5]. No alteration of a periplasmic or cytoplasmic membrane component is necessary to accommodate EDDHA.

Utilization of ferric siderophores requires the ferrous iron cytoplasmic membrane transporter FeoAB [5,7]. This means that iron must be reduced to the ferrous form and dissociate from the siderophore in the periplasm. Thus, the ferric siderophore does not enter the cytoplasm intact, and the transport of iron from the siderophore into the cytoplasm is not limited to a specific siderophore.

The iron moiety of the ferric siderophore is reduced to ferrous iron and dissociated from the siderophore in the periplasm by the promiscuous siderophore reductase FsrB [8]. The free ferrous iron is the substrate for FeoAB. Thus, the activities of FsrB and FeoAB can explain why siderophore selectivity is confined to the outer membrane. This is underscored by the observation that utilization of synthetic ferric EDDHA as an iron source requires FsrB and FeoAB, and only mutation of an outer membrane receptor gene is required. Utilization of inorganic ferric iron requires FeoAB, showing that it must be reduced prior to transport into the cytoplasm [7]. However, utilization of inorganic iron is independent of FsrB. Thus, a different, unknown ferric reductase is required to reduce inorganic iron [8].

We have previously observed that spontaneous mutations of outer membrane transporter genes can accommodate novel iron sources in iron starvation. These mutations occur at a frequency of about 10^−7^ [5]. Because *B*. *japonicum* does not synthesize siderophores, we speculate that this high frequency of mutation may be an adaptive response to acquire novel iron chelates within microbial communities. FsrB is a key to this strategy because it is non-selective for siderophores.

FsrB is a member of the COG3182 protein family, which includes the *Pseudomonas aeruginosa* ferric reductase FoxB [9]. Unlike FsrB, FoxB is reported to have a narrow substrate specificity, recognizing the closely related siderophores ferrioxamine B and ferrioxamine E [9]. The structural basis for this difference in specificity is currently unknown.

In the current study, we further analyze siderophore metabolism in *B*. *japonicum* by selection and characterization of *fsrB* mutant suppressor strains with restored ability to grow on ferric siderophores. We identified genes encoding export proteins that maintain homeostasis in the periplasm.

## Materials and methods

### Strains and media

All strains in this study are derived from *Bradyrhizobium japonicum* USDA110 (Table 1). Strains were routinely grown at 29°C shaking at 150 rpm in glycerol-salt-yeast (GSY) medium (0.3 g/liter KH_2_PO_4_, 0.3 g/liter Na_2_HPO_4_, 0.1 g/liter MgSO_4_-7H_2_O, 0.05 g/liter CaCl_2_-2H_2_O, 4 g/liter glycerol, 1 g/liter yeast extract, 0.01 g/liter H_3_BO_3_, 0.001 g/liter ZnSO_4_-2H_2_O, 0.001 g/liter FeCl_3_-6H_2_O, 0.0005 g/liter CuSO_4_-5H_2_O, 0.0005 g/liter MnCl_2_-4H_2_O, 0.0005 g/liter Na_2_MoO_4_-2H_2_O, 0.0001 g/liter biotin, pH 6.8). Low iron media was prepared as ½GSY, containing 0.5 g/liter of yeast extract with no added FeCl_3_. Where indicated, ½GSY media were also supplemented with siderophore to a final concentration of 25 μM ferrioxamine B or 4 μM ferrichrome.

**Table 1:** *B*. *japonicum* strains used in this study.

Strain	Description	Source
*B*. *japonicum* USDA110	Wild-type parent strain	USDA collection
*fsrB*	Δ*4505*	Ong, 2023
*fegA*	*blr4920*::Ω	Chatterjee, 2018
*feoB*	Δ*blr6523*	Sankari, 2016
*exsF*	Δ*blr2860*	This study
*exsG*	Δ*blr2861*	This study
*fsrB exsF*	Δ*blr4505* Δ*blr2860*	This study
*fsrB exsG*	Δ*blr4505* Δ*blr2861*	This study
*exsG feoB*	Δ*blr2861* Δ*blr6523*	This study
*fsrB exsG feoB*	Δ*blr2861* Δ*blr6523 Δ4505*	This study

### Identification of suppressor mutants

Cells of the *fsrB* mutant were grown in liquid GSY to mid-log phase, then washed twice in iron-free PBS and resuspended in GSY medium. A total of 10^8^ cells were spread on ½GSY medium containing either 25 μM ferrioxamine B or 4 μM ferrichrome. The plates were incubated for 16–21 days at 29°C until colonies were observed. All colonies were picked and screened for the ability to grow on media containing siderophore through a spotting assay, described below. Suppressor mutants were designated D# for colonies picked from ferrioxamine B plates and F# from ferrichrome plates.

Genomic DNA (gDNA) of the parent strain and selected suppressor mutants were isolated using phenol-chloroform extraction. Whole genome sequences of the strains were determined by high-throughput sequencing using a NovaSeq 6000 (UB Genomics and Bioinformatics Center). Genomes of the suppressor mutants were compared to parent strain and mutations were tabulated in S1 Table. Mutations were confirmed using PCR and subsequent DNA sequencing of the PCR product (Roswell Park DNA Sequencing Lab).

### Construction of strains and plasmids

To generate deletion mutants, fragments with 500 bp flanking sequence were amplified from genomic DNA isolated from USDA110. These fragments were cloned into pBluescript SK+ in *E*. *coli*, and inverse PCR was performed to create the in-frame deletion. The resulting fragment was sub-cloned to the suicide vector pLO1, then incorporated into the parent strain via conjugation and selective recombination. Double recombinants were confirmed through growth assays on selective media followed by DNA sequencing around the deleted region.

### Spotting assay

Cultures of *B*. *japonicum* were grown in liquid ½GSY-Fe media to mid-log phase, then washed twice and resuspended in iron-free 1X PBS pH 7.4. The washed cells were normalized to an OD_540_ of 0.2, then serial diluted. 5 μL of each dilution (10^−2^ to 10^−6^) was spotted onto ½GSY agar containing the indicated iron sources. Growth was assessed visually.

### Iron uptake assay

Cultures of *B*. *japonicum* were grown in liquid ½GSY-Fe media to mid-log phase, then washed twice and normalized to an OD_540_ of 0.3 in 5 mL uptake buffer (0.2M MOPS, 2% glycerol, pH 6.8). Where indicated, uptake buffer was also supplemented with siderophore to a final concentration of 25 μM ferrioxamine B or 4 μM ferrichrome. Cells were shaken in a 29°C water bath for 30 min. At t = 0, ^55^Fe was added to a final concentration of 1 μM. At various time points, 1 mL aliquots were removed and quenched in ice-cold quenching buffer (0.1M Tris, 0.1M succinate, 10mM EDTA, pH 6.0), then passed through 0.45 mm membrane filter disks for collection. Disks were mixed with 3 mL scintillation fluid and counted on a Beckman Coulter LS 6500 scintillation counter. Internalized ^55^Fe levels were normalized to protein content per sample.

### Antibiotic sensitivity assays

½GSY agar was prepared as described above. Where indicated, liquid and agar medium contained 0.2 μM or 0.5 μM iron-free albomycin, respectively. For tetracycline sensitivity assays, tetracycline stock solutions were prepared in 50% ethanol and diluted in growth medium to a final titration of 5, 10, or 20 μg/mL.

Antibiotic sensitivity was determined in both liquid and agar medium. Cells were grown to mid-log phase, then washed and resuspended in iron-free PBS. 10^6^ cells of *B*. *japonicum* were inoculated in liquid ½GSY-Fe media and grown at 29°C. Growth in liquid was assessed by measuring the OD_540_ of the cultures. Growth on plates was performed as described above.

## Results

### Selection for secondary site mutations that suppress the growth phenotype of the *fsrB* mutant

We previously identified FsrB as a ferric siderophore reductase that is required for utilization of siderophores as iron sources [8]. FsrB activity occurs in the periplasm, and reduction of iron is likely sufficient for its dissociation from the siderophore. Ferrous iron is then transported into the cytoplasm by FeoAB. To further characterize siderophore metabolism, we selected for secondary site mutations that suppress the *fsrB* phenotype.

The *fsrB* mutant does not grow on the siderophores ferrioxamine B or ferrichrome as the sole iron sources [8]. Cells of the mutant were spread onto agar plates containing either 25 μM ferrioxamine B or 4 μM ferrichrome, and colonies arising spontaneously after 16 to 21 days of incubation were re-streaked on the same respective medium to confirm the growth phenotype. 15 and 13 suppressor mutants that grew on ferrioxamine B and ferrichrome, respectively, were genotyped by whole-genome sequencing, along with the wild type and *fsrB* strains. Mutations in the suppressor strains were confirmed by PCR amplification of the regions around the lesions followed by DNA sequencing. A list of identified suppressor mutations is detailed in S1 Table.

Interestingly, all the suppressor strains harbored mutations within one of the three genes *blr2860*, *blr2861*, or *blr4473* (S1 Table). These genes are predicted to encode members of an RND efflux pump assembly and have been named these genes *exsF*, *exsG*, and *exsH*, respectively. RND efflux pumps are tripartite systems involved in the export of a wide range of substrates and are common in gram-negative bacteria [10–12]. *exsG* and *exsH* encode the inner membrane efflux transporter and the outer membrane TolC-family channel, respectively, and *exsF* encodes a periplasmic fusion “adaptor” protein that bridges the inner and outer membrane components. ExsF, ExsG and ExsH model well with the respective known structures of RND efflux proteins (S1 Fig). *exsF* and *exsG* are adjacent genes separated by 74 nucleotides within the *B*. *japonicum* genome, and they are predicted to not be co-transcribed in an operon (www.microbesonline.org). The genes on either side of *exsH* are greater than 200 nucleotides away, and it is predicted to not be co-transcribed with other genes.

The mutations harbored within the *exsF*, *exsG* and *exsH* genes included single nucleotide variations and indels, many of which resulted in frameshift mutations and premature termination codons (S2 Table). Therefore, they are most likely loss-of-function mutations. To further address this, we constructed mutants with in-frame deletion in the *exsF* or *exsG* gene and tested their ability to suppress the *fsrB* mutant growth phenotype. We were unable to construct an *exsH* deletion strain, suggesting that that the suppressor alleles retain some activity necessary for viability.

**Fig 1:**
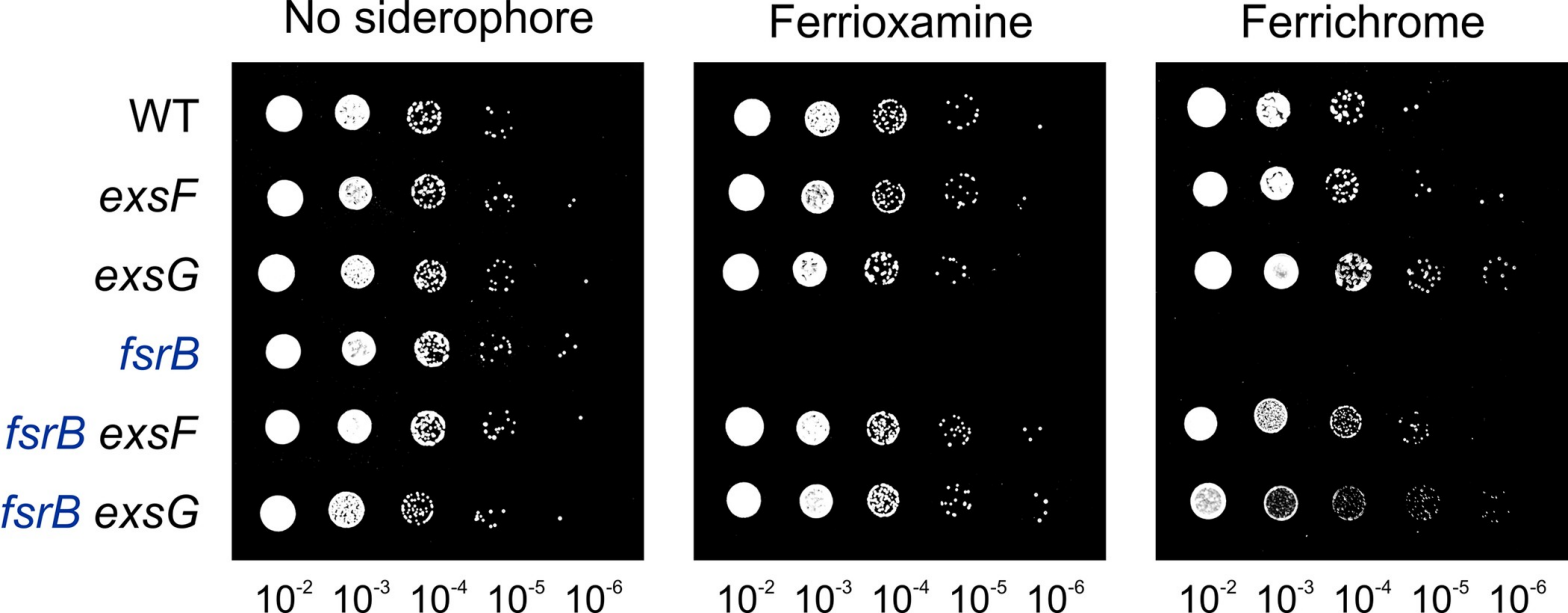
Deletion of *exsF* or *exsG* rescue the growth phenotype of *fsrB*. Cells of the wild type, *fsrB*, *exsF* (*blr2860*), *exsG* (*blr2861*), *fsrB exsF*, and *fsrB exsG* strains were grown in liquid medium, serial diluted 10^−2^ to 10^−6^, and 5 μL aliquots of each dilution were spotted on agar medium containing either no added siderophore, 10 μM ferrioxamine B, or 4 μM ferrichrome.

Like the original suppressor alleles, the *exsF* and *exsG* deletion strains suppressed the *fsrB* growth phenotype on ferrioxamine B or ferrichrome, as observed in a spotting assay (Fig 1). Thus, suppression of the *fsrB* mutant involves loss of function of *exsF* and *exsG*. FsrB was shown previously to act on structurally diverse ferric siderophores [8]. The fact that *exsF*, *exsG* and *exsH* were each identified in two independent selection protocols, one on ferrioxamine B and the other on ferrichrome, indicates that their gene products act on structurally disparate compounds. Similarly, the *exsF* and *exsG* deletion strains rescue the growth of the *fsrB* mutant on both ferrioxamine B and ferrichrome. *exsF* and *exsG* deletion strains were also constructed in a wild type (*fsrB*^+^) background and those single mutants showed no growth phenotype on media containing ferric siderophore as an iron source (Fig 1). Neither the single nor double mutants showed a growth phenotype on inorganic iron.

### Mutation of the *exsG* gene restores accumulated iron levels in the *fsrB* mutant background

The *fsrB* mutant is iron-deficient when ferric siderophore is the iron source [8], and thus it does not grow under those conditions. Because the *exsF*, *exsG*, and *exsH* genes encode export proteins, it is likely that the uptake deficiency by the *fsrB* strain is compensated by loss-of-function of export activity.

**Fig 2:**
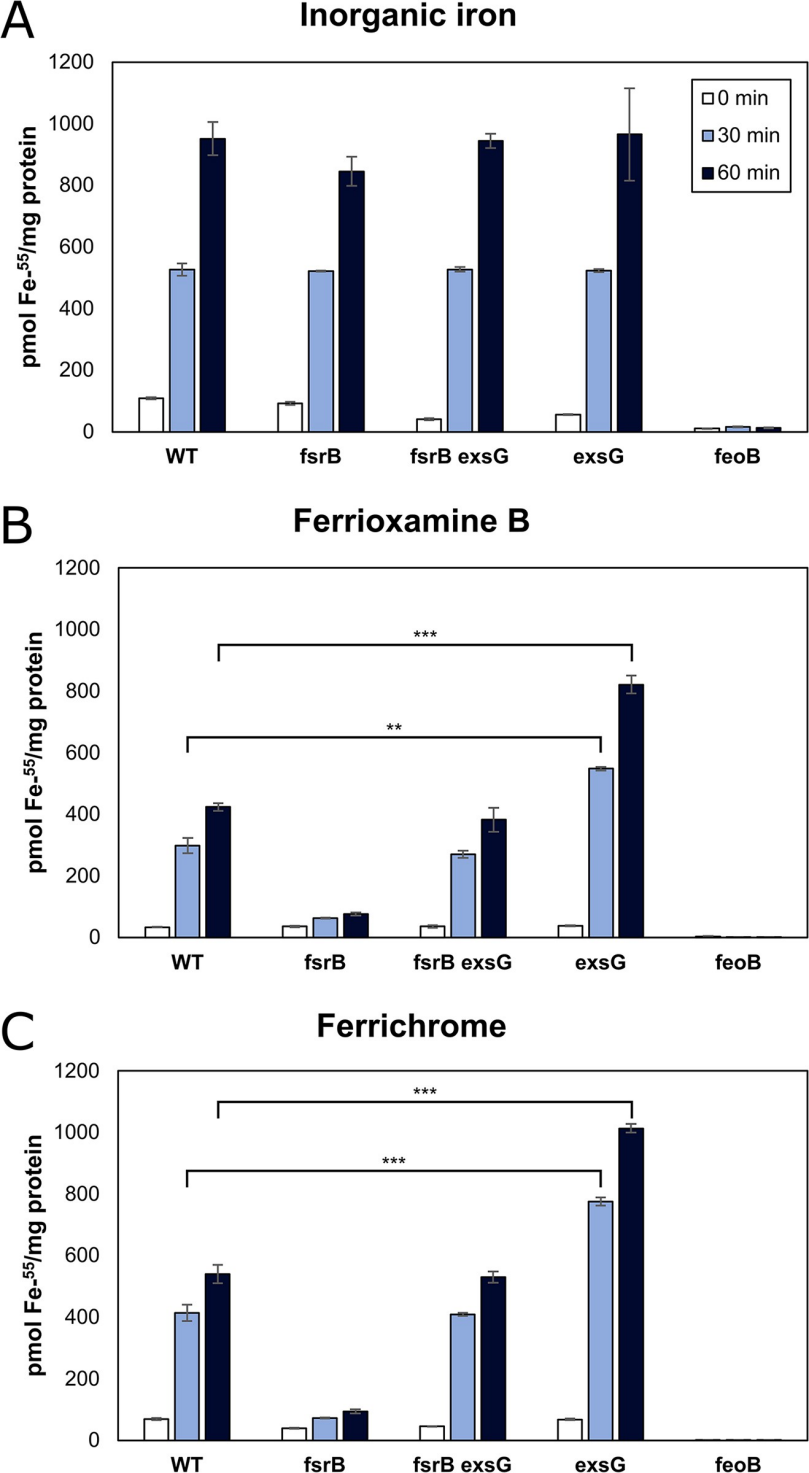
Accumulation of ^55^Fe from ferric siderophores by wild type and mutant cells. Wild type, *fsrB*, *fsrB exsG*, *exsG* and *feoB* cells were grown in liquid culture, then resuspended in uptake buffer. (A) 1 μM ^55^Fe was added to the reaction at t = 0. Cells were harvested at t = 0, 30, and 60 min, collected on membrane filter disks and radioactive signal was counted. Where indicated, ^55^Fe was chelated with (B) 25 μM desferrioxamine B or (C) 4 μM desferrichrome prior to reaction. Data are represented as picomoles ^55^Fe taken up per milligram of protein ± the standard deviation. (***) *p* < 0.001, (**) *p* < 0.01 (Student’s *t*-test).

To test this hypothesis, we compared ^55^Fe accumulation from ferrioxamine B or ferrichrome in cells of the wild type, *fsrB* and *fsrB exsG* strains (Fig 2). Whereas the ^55^Fe iron levels were unaffected in the *fsrB* mutant with an inorganic iron source compared to wild type levels (Fig 2A), they were much lower with ^55^Fe-siderophore as the iron source (Fig 2B and 2C). This agrees with previous findings that FsrB is a periplasmic ferric siderophore reductase [8]. ^55^Fe iron levels are higher in the *fsrB* mutant than in the *feoB* strain, suggesting a low level of iron assimilation in the *fsrB* mutant.

Mutation of the *exsG* gene in an *fsrB* background restored iron to approximately wild type levels (Fig 2B and 2C), which is consistent with the rescued growth of the *fsrB* mutant on siderophore by mutation of *exs* genes (Fig 1). We suggest that iron levels are low in the *fsrB* mutant because iron is not reduced or dissociated from siderophore, resulting in export of the ferric siderophore from the periplasm by ExsFGH.

Mutation of *exsG* in a wild type (*fsrB*^+^) background resulted in higher ^55^Fe levels with ferric ferrioxamine B (Fig 2B) or ferrichrome (Fig 2C) as substrate than was found in the wild type (Fig 2B and 2C), which is consistent with a fully functional iron assimilation process, but with a defect in export. ^55^Fe levels were unaffected by the *exsG* mutation with inorganic iron as substrate, showing that ExsFGH is not an exporter of inorganic iron. This latter result also agrees with our previous findings that inorganic iron is exported by a different exporter [13].

### The rescued *exsG fsrB* mutant requires *feoB* for growth

We previously showed that utilization of iron from ferric siderophore involves reduction of the iron moiety and dissociation from the siderophore via FsrB in the periplasm [8], followed by transport of the ferrous iron into the cytoplasm by the FeoAB transport system [5,7]. If the export deficiency created by *exsG* gene mutation in the *fsrB* background allowed ferric siderophore to accumulate in the periplasm and enter the cytoplasm intact by an undescribed siderophore transporter, we would expect that the rescue of the *fsrB* mutant by *exsG* deletion would be independent of *feoB*. However, if loss of *exsG* in the *fsrB* mutant allowed iron reduction in the periplasm, then growth of the rescued double mutant would still require *feoB*.

**Fig 3:**
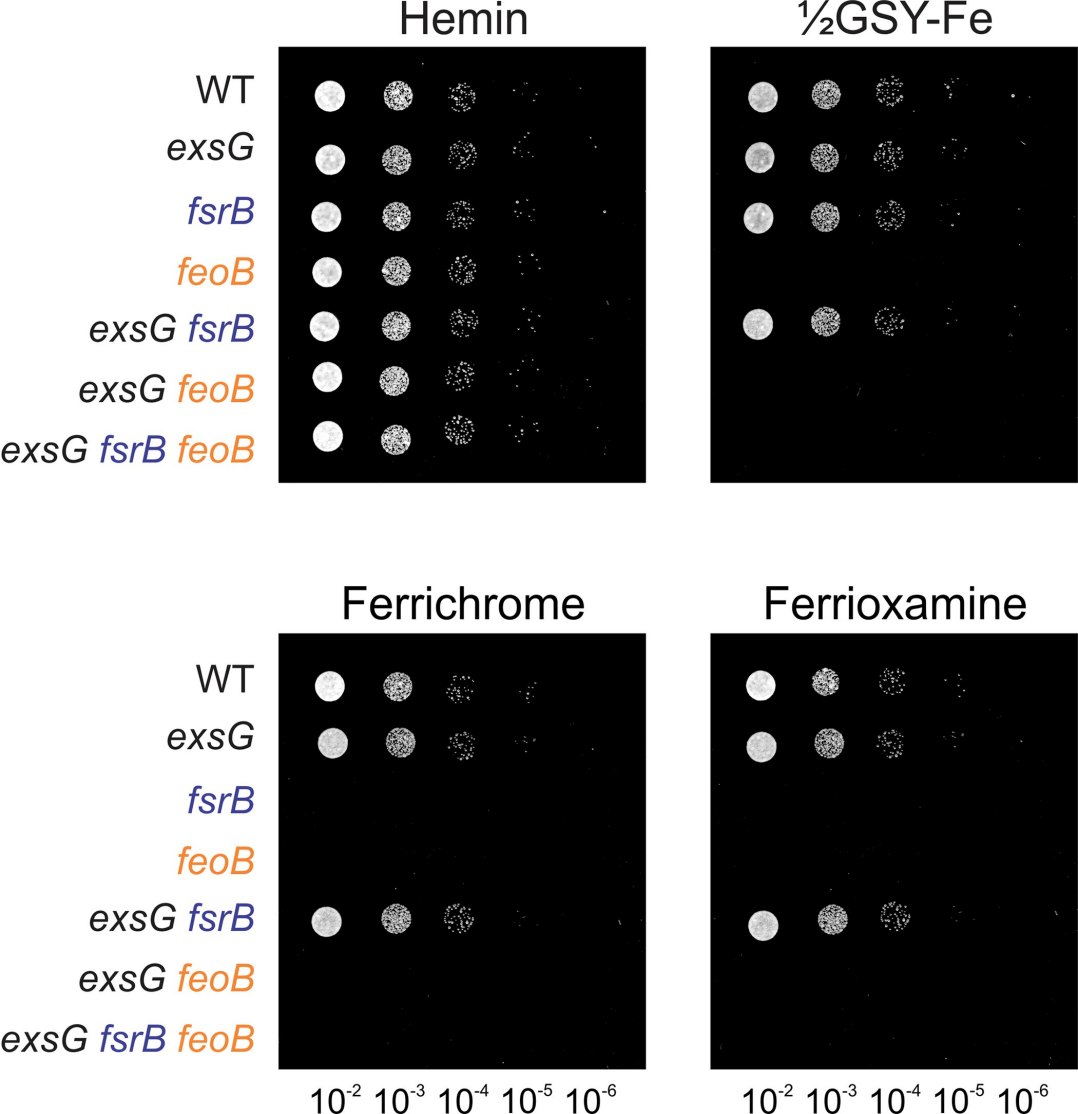
*feoB* is required for growth of the *fsrB exsG* mutant. Cells of the wild type, and mutant constructs of *exsG*, *fsrB*, *feoB*, and double and triple mutants were grown, diluted, and then spotted as described in Fig 1 legend on agar containing either no added iron, 4 μM ferrichrome, 25 μM ferrioxamine B, or 10 μg/mL hemin.

To test this, we constructed various deletion mutants and compared their ability to grow on inorganic iron, ferrioxamine B, ferrichrome, and hemin (Fig 3). As previously described, the *feoB* mutant was defective in growth on media containing inorganic iron or siderophore but grew well on heme [5,7]. The *exsG* mutant grew on all the tested iron sources, but the *exsG feoB* double mutant behaved similarly to the *feoB* mutant. Likewise, although the *exsG fsrB* double mutant grew on siderophore, the *exsG fsrB feoB* triple mutant did not (Fig 3). Therefore, a deletion of *exsG* can compensate for the *fsrB* growth phenotype, but it still requires the inner membrane transporter FeoB for the utilization of ferric siderophores. This suggests that the function of the Exs system is to export siderophore from the periplasm.

### Deletion of *exsG* imparts sensitivity to albomycin

Albomycin is a naturally occurring sideromycin compound, consisting of the siderophore ferrichrome linked to an antibiotic moiety that inhibits tRNA synthetases [14,15]. In *E*. *coli*, albomycin enters the cytoplasm intact by the ferrichrome uptake system and is subsequently hydrolyzed there to exert its antibiotic activity [16].

*B*. *japonicum* is partially resistant to albomycin relative to *E*. *coli* because the major route of iron acquisition from siderophores does not require that the siderophore enter the cytoplasm in *B*. *japonicum* [5]. Partial sensitivity is conferred by the outer membrane ferrichrome receptor FegA and presumably an alternative inner membrane transporter [5].

**Fig 4:**
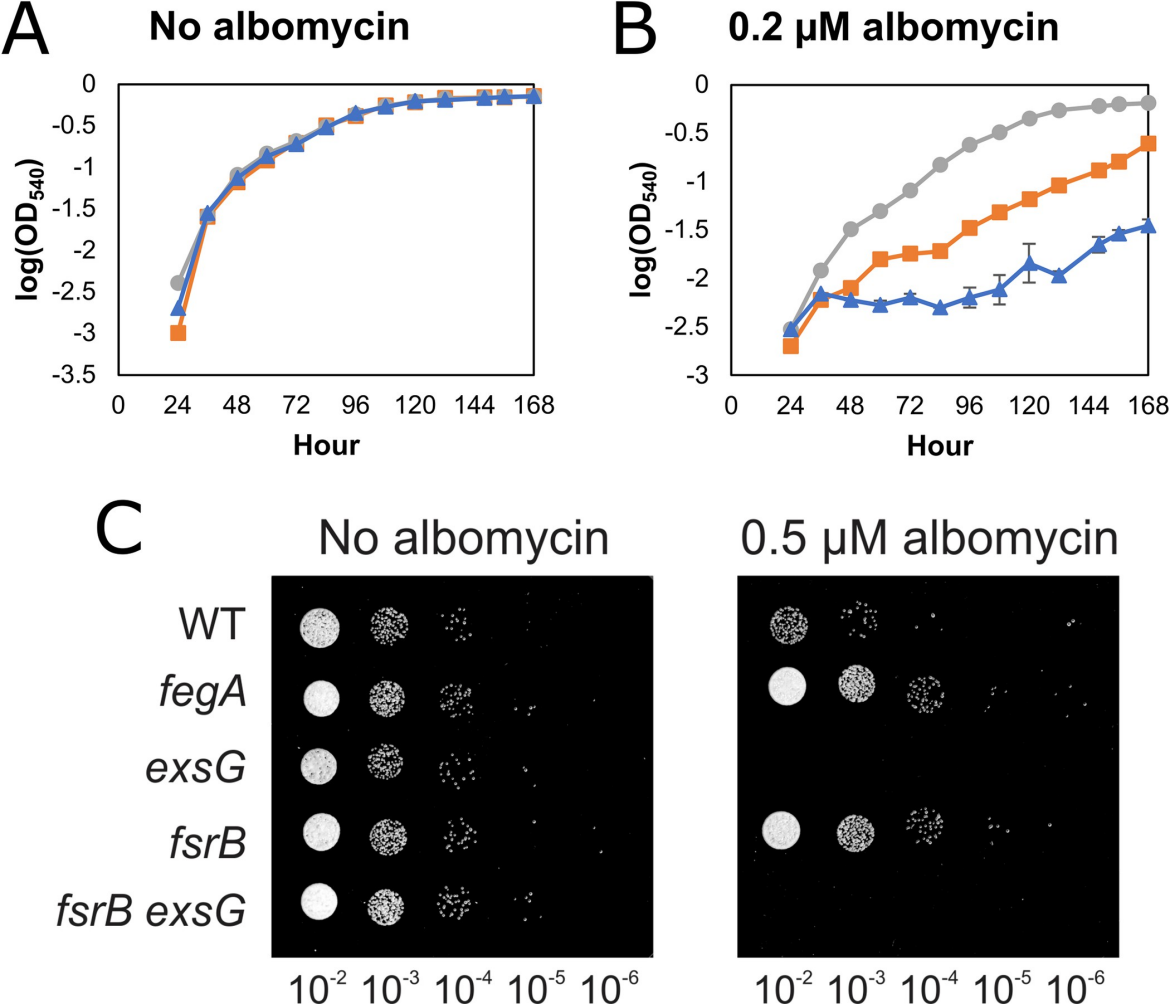
Deletion of *exsG* imparts albomycin sensitivity. Growth of the wild type (■), and *exsG* (▲) or *fegA* (●) mutants in low-iron liquid culture containing (A) no albomycin or (B) 0.2 μM albomycin (iron-free). Each time point is represented as an average of triplicate samples ± standard deviation. (C) Cells of the wild type, *fegA*, *exsG*, *fsrB*, and *fsrB exsG* strains were grown, diluted, and then spotted as described in Fig 1 legend on agar containing either 0 μM or 0.5 μM albomycin (iron-free).

We tested the *exsG* mutant for its sensitivity to albomycin to determine whether the Exs system plays a role in albomycin resistance. In the absence of albomycin, the wild type and the *fegA* and *exsG* mutants grew similarly in liquid culture (Fig 4A). With the addition of 0.2 μM albomycin, growth of the wild type was impaired, but growth of the *fegA* mutant was unimpaired (Fig 4A). Growth of the *exsG* mutant was more severely diminished than the wild type. The effects of albomycin on growth of the wild type and mutants were also tested on solid media in a spotting assay (Fig 4B). There, the *exsG* mutant also exhibited increased sensitivity to albomycin, and the *fegA* mutant was more resistant compared to the wild type.

The *fsrB* mutant showed increased resistance to albomycin similar to that observed for the *fegA* mutant. Because FsrB functions in the periplasm to reduce ferrous iron leading to its dissociation from the siderophore, the increased resistance of the *fsrB* mutant to albomycin indicates that ExsFGH exports ferrated albomycin from the periplasm.

In these experiments, wild type *B*. *japonicum* has partial sensitivity to albomycin that is made more sensitive by blocking export. Therefore, there must be a low-activity inner membrane transporter that allows the albomycin to enter the cytoplasm but is insufficient to support growth on the siderophores ferrioxamine or ferrichrome. It is not yet clear what this mechanism is.

### Deletion of *exsG* confers sensitivity to the antibiotic tetracycline

**Fig 5:**
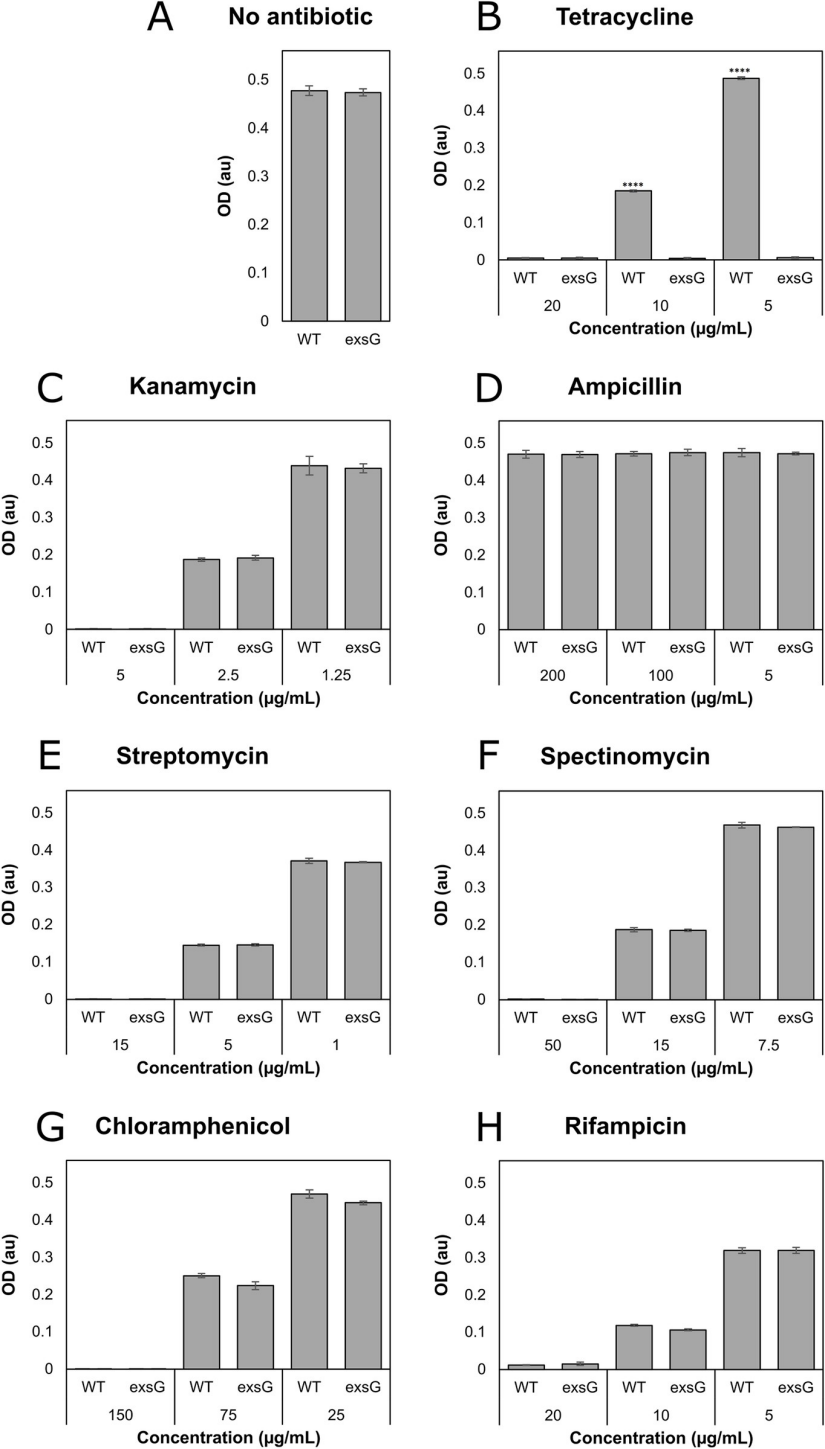
The *exsG* deletion confers sensitivity to tetracycline. Cells of the wild type and the *exsG* strain were grown a 96-well plate in GSY medium containing antibiotics with the indicated concentrations (μg/mL): A) no antibiotic, (B) tetracycline (20/10/5), (C) kanamycin (5/2.5/1.25), (D) ampicillin (200/100/5), (E) streptomycin (15/5/1), (F) spectinomycin (50/15/7.5), (G) chloramphenicol (150/75/25), (H) rifampicin (20/10/5). Growth is represented as the OD_540_ after 3 days ± SD. No significant difference was observed between the wild type and *exsG* strain in any condition except tetracycline. (****) *p* < 0.0001 between the wild type and the *exsG* mutants at 5 μg/ml and 10 μg/ml tetracycline.

*B*. *japonicum* is resistant to numerous antibiotics [17], and some RND family transporters export a wide range of substrates, and some exporters confer resistance to antibiotics [18]. Therefore, we wanted to test whether the Exs system contributed to resistance to antibiotics in *B*. *japonicum* in addition to the siderophore antibiotic albomycin. We screened several antibiotics for growth in liquid medium and observed that the *exsG* mutant was more sensitive than the wild type to tetracycline (Fig 5), indicating that ExsFGH contributes to tetracycline resistance in wild type cells.

**Fig 6:**
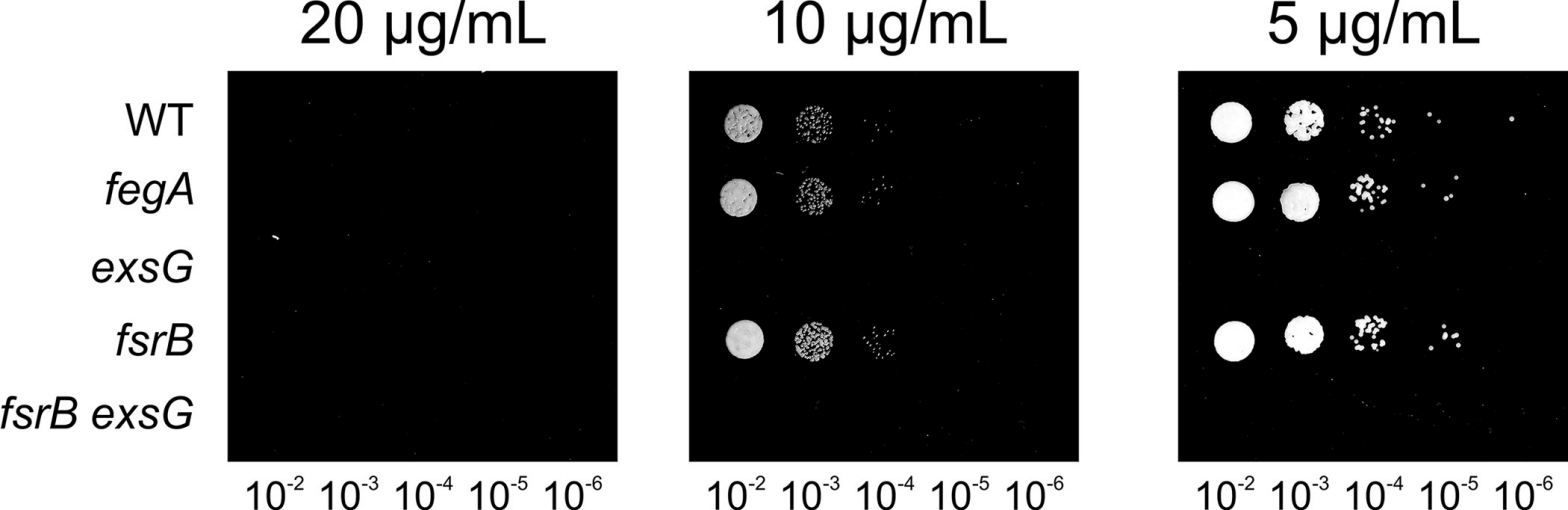
Effect of tetracycline on growth of wild type and mutants on solid agar media. Cells of the wild type, *fegA*, *exsG*, *fsrB*, and *fsrB exsG* strains were grown, diluted, and then spotted as described in Fig 1 legend on agar containing 20, 10, or 5 μg/mL tetracycline.

We also examined the effects of growth in the presence of tetracycline on solid agar media and included additional mutants (Fig 6). Growth of the wild type was completely inhibited on plates containing 20 μg/mL tetracycline, but it tolerated lower concentrations. Similar to liquid growth, the *exsG* mutant was highly sensitive to tetracycline at 5 mg/ml or 10 mg/ml of the antibiotic, as was the *fsrB exsG* double mutant.

Unlike albomycin, the *fegA* mutant was not more resistant to tetracycline compared to the wild type. In addition, mutation of the *fsrB* gene also had no effect on tetracycline sensitivity. These findings are not surprising because tetracycline transport across the outer and inner bacterial membranes differ from siderophore-mediated uptake [19].

## Discussion

**Fig 7:**
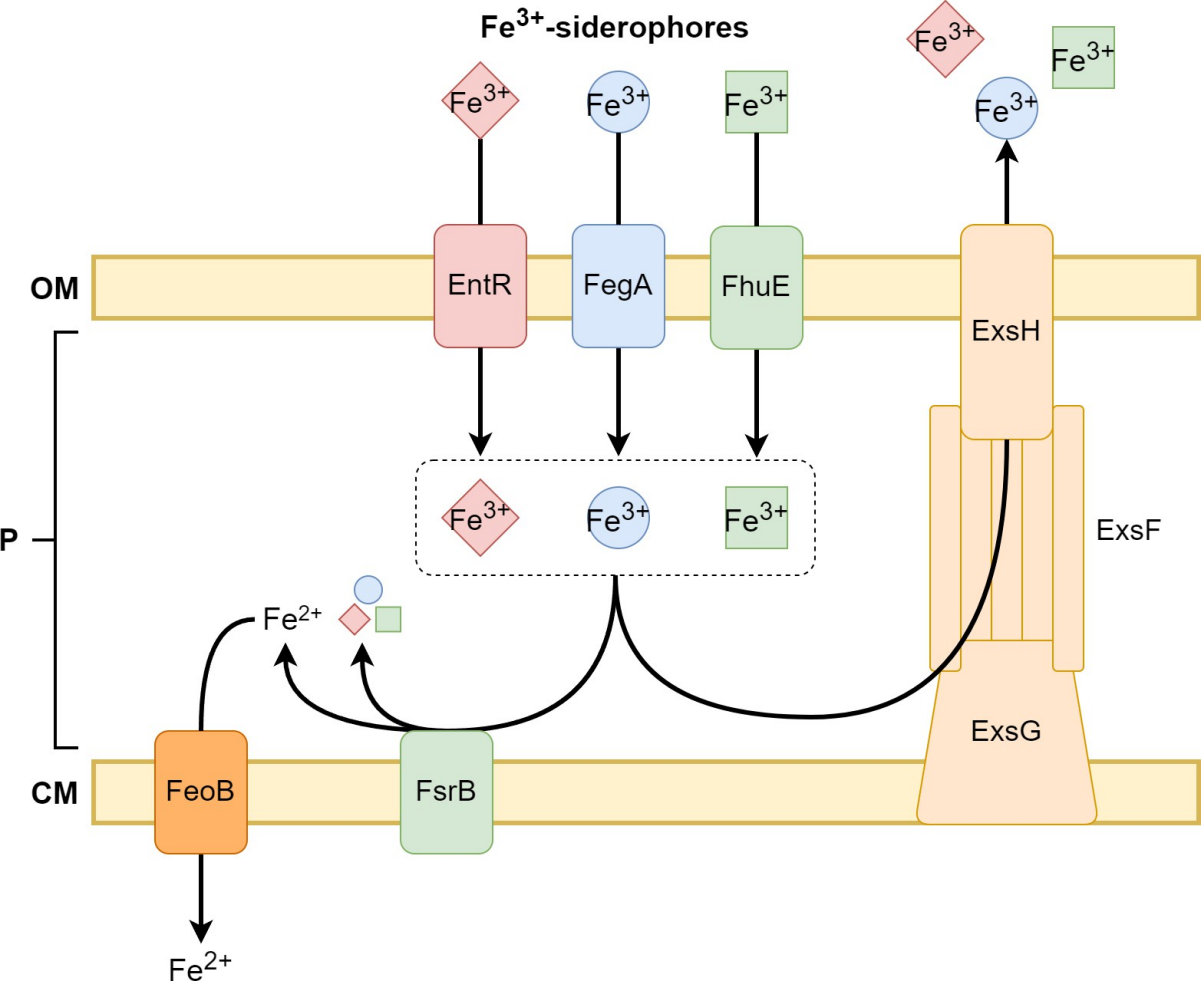
Ferric siderophore utilization and export in the periplasm. Proposed model of ferric siderophore uptake in *B*. *japonicum*. Various ferric siderophores are transported across the outer membrane through their corresponding receptors. In the periplasm, the ferric siderophores are either metabolized through FsrB or exported through the ExsFGH complex. The fate of the deferrated siderophores are not known.

In this study, we identified *exsF*, *exsG* and *exsH* as exporter genes involved in maintaining homeostasis of iron acquired from environmental ferric siderophores, but not from inorganic iron. Our findings support a model whereby ferric siderophores enter the periplasm via an outer membrane receptor, followed either by metabolism of the siderophore initiated by FsrB or export from the periplasm via the ExsFGH efflux system (Fig 7). Thus, the periplasm is a regulated compartment with respect to maintaining ferric siderophore levels. The need for siderophore efflux may depend on its availability in the environment and also on other iron sources that may be concurrently available. It may also depend on the flux of electrons available to FsrB. For example, cells may have access to both siderophore iron and inorganic iron or may have a low availability of reductant to reduce siderophore iron by FsrB. These situations may require ferric siderophore efflux from the periplasm.

X-ray crystal structures of RND family transporters indicate that they export solutes from the cytoplasm and the periplasm [20–22]. Because ferric siderophores are metabolized in the periplasm in *B*. *japonicum* [5,8], the current study indicates that ExsFGH exports them from that compartment. The increased resistance of the *fsrB* mutant to albomycin also suggests that ExsFGH exports it from the periplasm. However, both albomycin and tetracycline exert their antibacterial activity in the cytoplasm, and thus ExsFGH may export those compounds from the cytoplasm as well.

Mutations in the *exs* genes suppress the growth phenotype of the *fsrB* mutant, but the rescued growth remains dependent on *feoB* as seen by the lack of growth of the *fsrB exsG feoB* triple mutant on siderophore (Fig 3). These findings strongly suggest that the iron must still be reduced and dissociated from the siderophore in the periplasm, prior to entry into the cytoplasm via FeoB. Thus, our model suggests that there exists a yet to be described reductase which can metabolize siderophores in the periplasm when the siderophore concentration in that compartment is elevated. However, the *fsrB* mutant cannot grow on siderophores [8] (Fig 1), and therefore the undescribed reductase likely has low affinity for siderophore that cannot compensate for the *fsrB* lesion unless the periplasmic siderophore concentration is high. We have not identified the alternative reductase, nor is it likely to play a role in siderophore metabolism in wild type cells under growth conditions that we routinely employ.

Although wild type *B*. *japonicum* is more resistant to the antibiotic siderophore albomycin compared with *E*. *coli* [5], it does display sensitivity to it relative to a *fegA* mutant (Fig 4). Thus, albomycin must enter the cytoplasm to exert its antibiotic activity. This observation is not in conflict with the conclusion that ferric siderophores do not enter the cytoplasm intact to fulfill the cellular iron requirement. This is because the quantity of albomycin sufficient to poison its cytoplasmic target is likely much lower than the quantity of siderophore that would need to enter the cytoplasm to support growth. Thus, small quantities of non-toxic siderophores that may enter the cytoplasm would not support growth, consistent with the requirement for *fsrB* and *feoB* to utilize them.

Our data support the conclusion that the ExsFGH system exports the ferrated form of the siderophore. Firstly, ExsFGH confers resistance to albomycin, and the *fsrB* mutant is more resistant to albomycin than is the wild type (Fig 4). This finding indicates that ExsFGH confers resistance by exporting the ferrated form of albomycin. Secondly, the *exsG* strain accumulates more iron than the wild type (Fig 2), which we would not expect if ExsFGH did not normally expel the iron-bound siderophore from the cell. In principle, this could be an indirect effect of a defect in export of deferrated siderophore, but efflux of the ferrated form is the most parsimonious explanation when taking the albomycin data into account.

In wild type *B*. *japonicum* cells, siderophores dissociate from iron in the periplasm, and so there must be a mechanism for removal of the deferrated molecule, or a derivative of it, from that compartment. Although ExsFGH very likely expels ferric siderophores, we cannot rule out that it exports the deferrated siderophore as well.

The *exsG* mutant was sensitive to the antibiotic tetracycline compared with the wild type, but not to other antibiotics (Fig 5). Although tetracycline does not have siderophore function, it is, to our knowledge, the only antibiotic tested that binds to iron and other divalent metals, with a logK_f_ for Fe^3+^ of 25.3 [23–25]. This raises the possibility that iron is an identifying feature for compounds recognized by ExsFGH. More work is needed to resolve this question.

The current study supports the conclusion that ExsFGH exports structurally diverse compounds (S2 Fig), which is consistent with the activities of other RND exporters. The *E*. *coli* ArcAB-TolC and *P*. *aeruginosa* MexAB-OprM exporters confer resistance to numerous structurally dissimilar antibiotics, and they pump out other non-antibiotic compounds as well [18,26]. A single RND heavy metal exporter can pump out numerous different divalent metals [13,27,28]. Thus, whereas import by gram negative bacteria typically proceeds by highly selective transporters, export is less stringent.

This important difference between uptake and export can be applied to formally rule out the idea that rescue of the *fsrB* growth phenotype by mutation of the *exs* genes resulted in a compensatory gain-of-function activity unrelated to an export defect. We postulate that the export defect in the *exs* mutants allows siderophore to accumulate in the periplasm so that a lower affinity reductase can act on it. Stimulation of an alternate reductase in the absence of export cannot explain the sensitivity of the *exsG* mutant to albomycin or tetracycline because those compounds are independent of *fsrB* (Figs 4 and 6). Similarly, a hypothetical upregulation of an outer membrane component in the *exsG* mutant to allow increased siderophore uptake into the periplasm would not explain sensitivity of the *exsG* mutant to tetracycline, which is not taken up by siderophore transporters (Fig 6) [19]. As RND transporters are known exporters with broad substrate selectivity, roles for ExsFGH in the efflux of ferrichrome, ferrioxamine, albomycin and tetracycline is consistent with all of the data presented here.

## Supporting information

S1 Fig Predicted structure of the RND assembly. ExsH (left, grey), ExsF (middle, blue), and ExsG (right, yellow) are modeled on the AcrAB-TolC RND efflux pump from E. coli (PDB 5V5S, https://doi.org/10.2210/pdb5V5S/pdb). ExsH is a homotrimeric TolC outer membrane protein; ExsG is a homotrimeric inner membrane RND efflux transporter; six subunits of ExsF join the inner and outer membrane components.
(TIF)

S2 Fig Structures compounds described in this study. (A) ferrichrome, (B) ferrioxamine B, (C) albomycin δ2, and (D) tetracycline are described with their iron coordination. Siderophore hydroxamate ligands are marked in red.
(TIF)

S1 Table List of suppressor mutants. Potential spontaneous suppressor mutants of the *fsrB* strain were selected on ferrioxamine B (denoted D) or ferrichrome (denoted F). Among them, 28 were successfully sequenced. 26 mutants carried a mutation in either *blr2860*, *blr2861*, or *blr4473*. One mutant (F42) contained a mutation in the *blr4473* promoter. Mutation type (Δ), nucleotide change (Δnt), and amino acid change (AAC) are detailed in the table. SNV, single nucleotide variation. NC, no amino acid change.
(TIF)

S2 Table Amino acid changes within suppressor mutants of the *fsrB* strain. (TIF)
