# Supplementary figures and images for: The Bradyrhizobium japonicum exporter ExsFGH is involved in efflux of ferric xenosiderophores from the periplasm
Source: PLoS One. 2024 Jan 2;19(1):e0296306. doi: 10.1371/journal.pone.0296306 (PMC10760861; doi:10.1371/journal.pone.0296306)

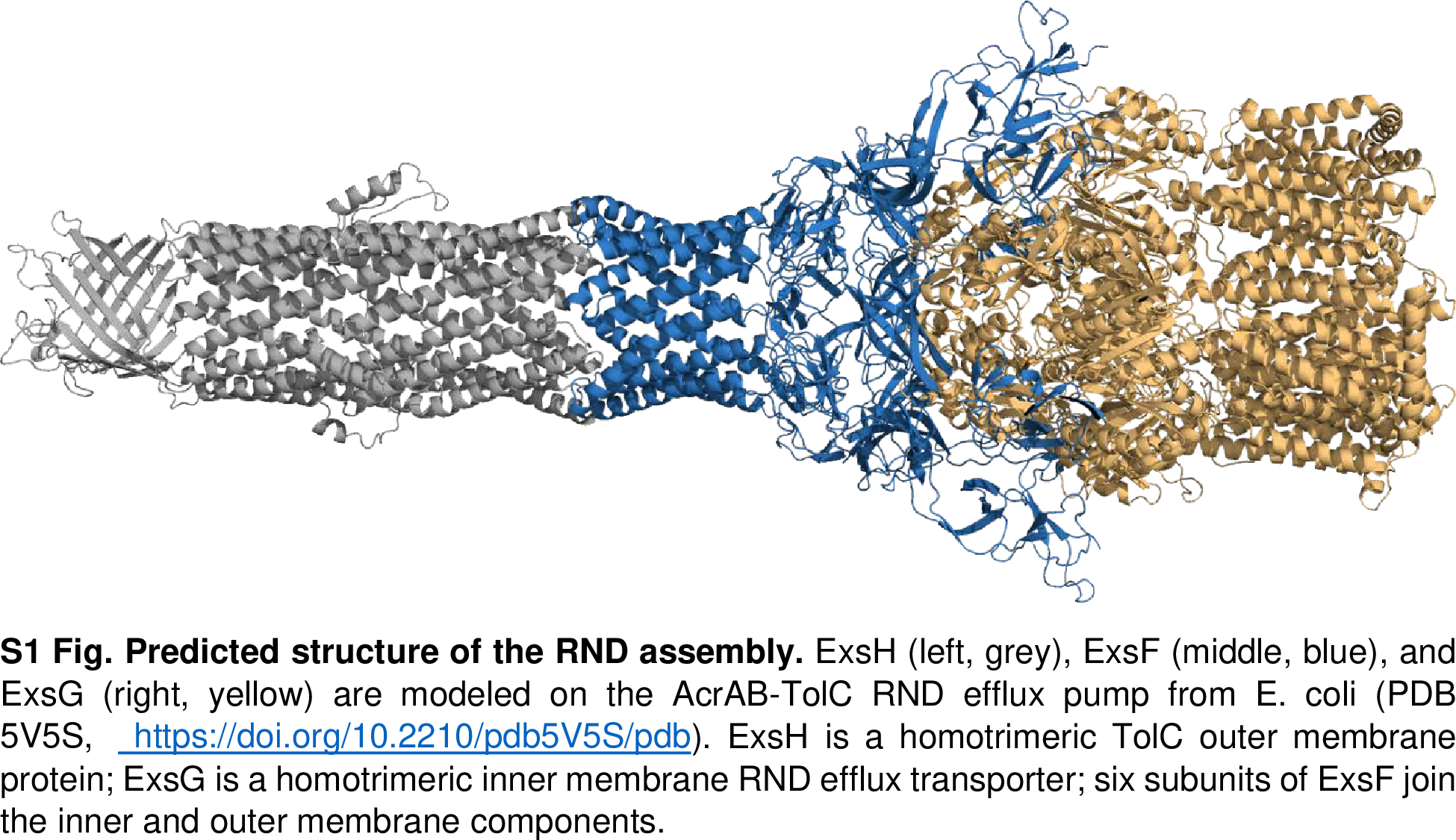

Supplement: S1 Fig — ExsH (left, grey), ExsF (middle, blue), and ExsG (right, yellow) are modeled on the AcrAB-TolC RND efflux pump from E. coli (PDB 5V5S, https://doi.org/10.2210/pdb5V5S/pdb). ExsH is a homotrimeric TolC outer membrane protein; ExsG is a homotrimeric inner membrane RND efflux transporter; six subunits of ExsF join the inner and outer membrane components. (TIF) [file pone.0296306.s001.tif]

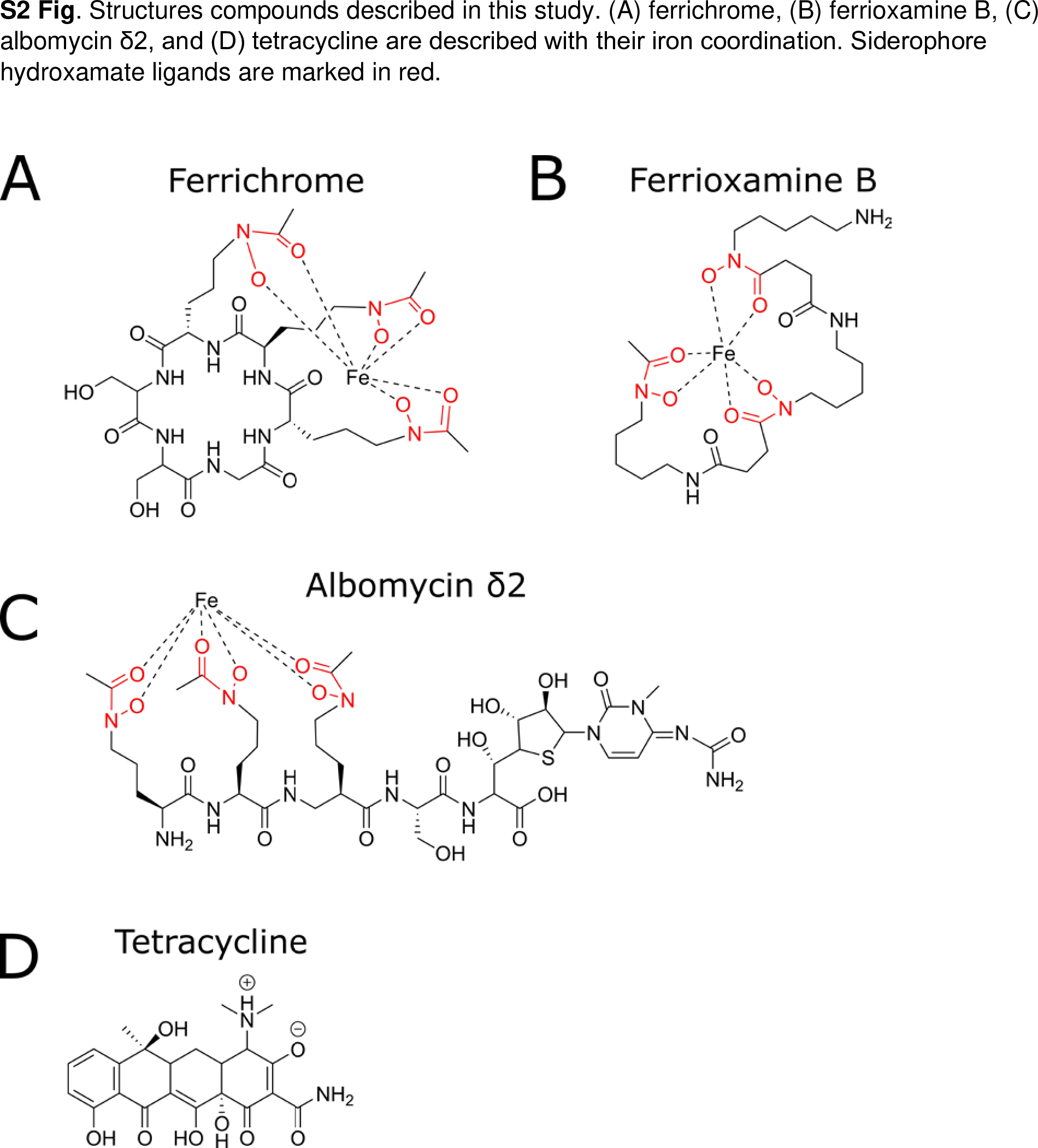

Supplement: S2 Fig — (A) ferrichrome, (B) ferrioxamine B, (C) albomycin δ2, and (D) tetracycline are described with their iron coordination. Siderophore hydroxamate ligands are marked in red. (TIF) [file pone.0296306.s002.tif]

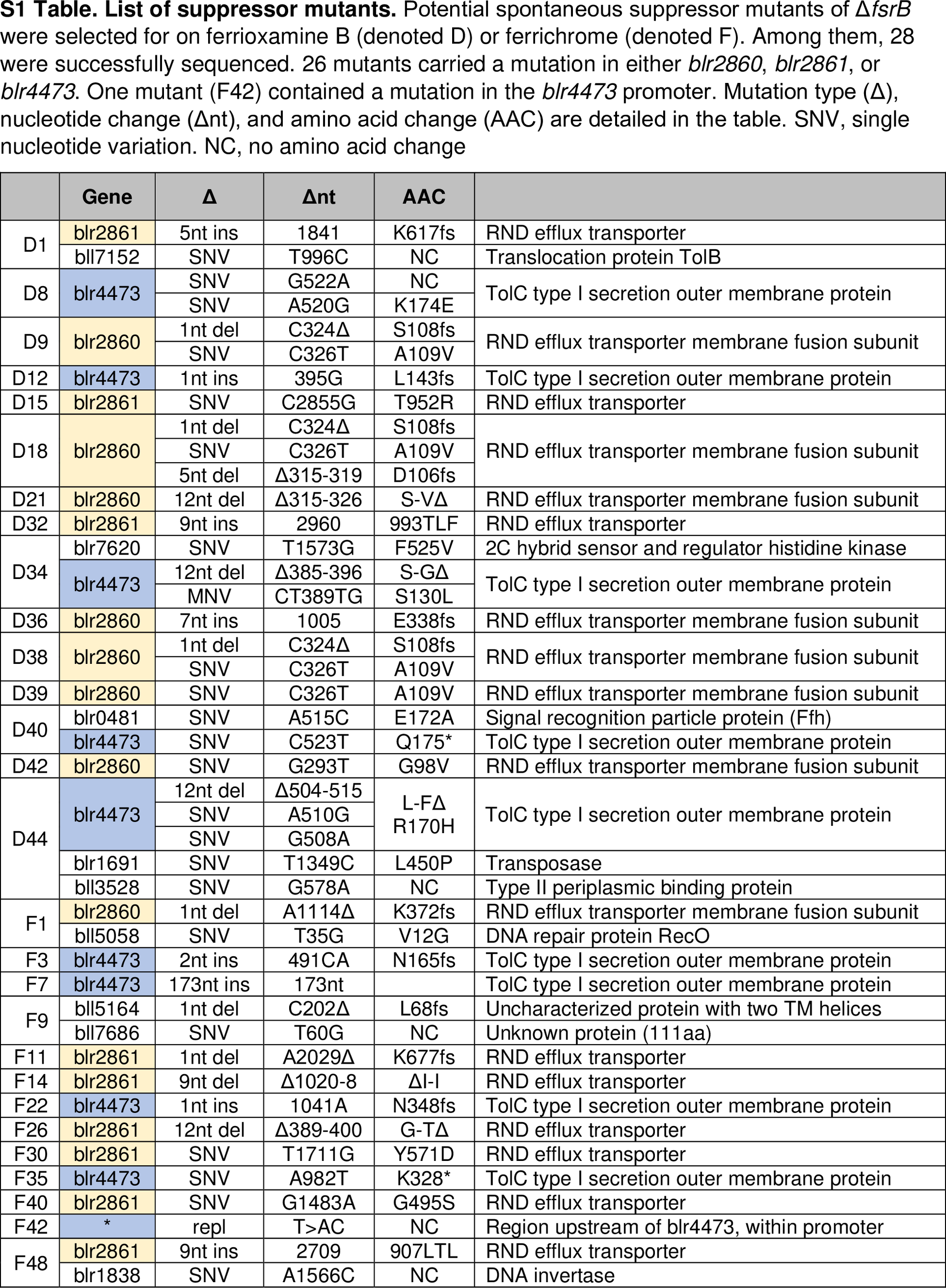

Supplement: S1 Table — Potential spontaneous suppressor mutants of the fsrB strain were selected on ferrioxamine B (denoted D) or ferrichrome (denoted F). Among them, 28 were successfully sequenced. 26 mutants carried a mutation in either blr2860, blr2861, or blr4473. One mutant (F42) contained a mutation in the blr4473 promoter. Mutation type (Δ), nucleotide change (Δnt), and amino acid change (AAC) are detailed in the table. SNV, single nucleotide variation. NC, no amino acid change. (TIF) [file pone.0296306.s003.tif]

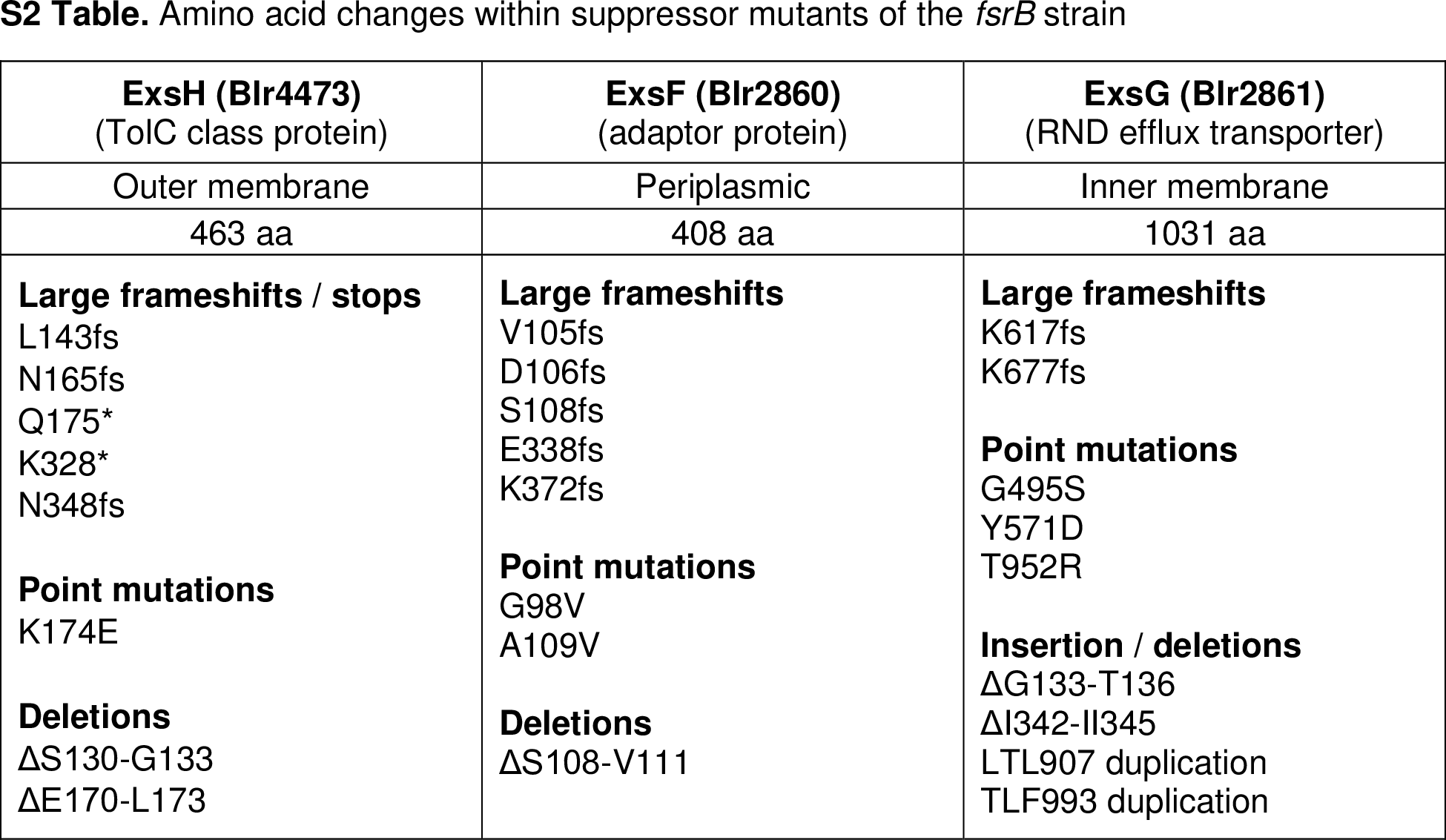

Supplement: S2 Table — (TIF) [file pone.0296306.s004.tif]
